# Supplementary material for: Symptomatic menopausal transition and risk of subsequent stroke
Source: PeerJ. 2019 Oct 30;7:e7964. doi: 10.7717/peerj.7964 (PMC6825412; doi:10.7717/peerj.7964)
Supplement: Supplemental Information 1 [file peerj-07-7964-s001.docx]

| **Variable** | **Description** | **Data Type** |
| --- | --- | --- |
| id | id |  |
| PScore | propensity score | continuous data |
| HF | Symptomatic menopausal transition | binary (1:yes 0:No) |
| INS_AMT | Monthly income, new Taiwan dollars | continuous data |
| age | Age, years | continuous data |
| CCI | Charlson’s comorbidity index score | continuous data |
| HPTbase | Hypertension | binary (1:yes 0:No) |
| hyperlipidbase | Hyperlipidemia | binary (1:yes 0:No) |
| dmbase | Diabetes mellitus | binary (1:yes 0:No) |
| obesitybase | Obesity | binary (1:yes 0:No) |
| ckdbase | chronic kidney disease | binary (1:yes 0:No) |
| cadbase | coronary artery disease | binary (1:yes 0:No) |
| chfbase | congestive heart failure | binary (1:yes 0:No) |
| copdbase | chronic obstructive pulmonary disease | binary (1:yes 0:No) |
| paodbase | peripheral artery occlusive disease | binary (1:yes 0:No) |
| Dysarrhythmiabase | Dysrhythmia | binary (1:yes 0:No) |
| antiHPTbase | Antihypertensive drugs | binary (1:user 0:nonuser) |
| dmdrugbase | Antidiabetic agents | binary (1:user 0:nonuser) |
| statinbase | statin | binary (1:user 0:nonuser) |
| hrtbase | Hormone replacement therapy | binary (1:user 0:nonuser) |
| estrogensbase | estrogens | binary (1:user 0:nonuser) |
| progestogensbase | progestogens | binary (1:user 0:nonuser) |
| prog_estrogensbase | Combined progestogens and estrogens | binary (1:user 0:nonuser) |
| aspirinbase | Aspirin | binary (1:user 0:nonuser) |
| Antiplateletbase | Antiplatelet | binary (1:user 0:nonuser) |
| Warfarinbase | Warfarin | binary (1:user 0:nonuser) |
| visit1yr | Clinic visit frequency, visits per year | continuous data |
| com_stroke | event status | category (1:stroke 2: death 0:censoring) |
| T | failure time | continuous data |
